# A parallel Canny edge detection algorithm based on OpenCL acceleration

**DOI:** 10.1371/journal.pone.0292345

**Published:** 2024-01-05

**Authors:** Yupu Song, Cailin Li, Shiyang Xiao, Qinglei Zhou, Han Xiao

**Affiliations:** 1 College of Computer Engineering, Shangqiu Polytechnic, Shangqiu, China; 2 School of Civil and Architectural Engineering, Shandong University of Technology, Zibo, China; 3 School of Civil Engineering, Southeast University, Nanjing, China; 4 School of Computer and Artificial Intelligence, Zhengzhou University, Zhengzhou, China; 5 School of Information Science and Technology, Zhengzhou Normal University, Zhengzhou, China; National University of Sciences and Technology NUST, PAKISTAN

## Abstract

In the process of Canny edge detection, a large number of high complexity calculations such as Gaussian filtering, gradient calculation, non-maximum suppression, and double threshold judgment need to be performed on the image, which takes up a lot of operation time, which is a great challenge to the real-time requirements of the algorithm. The traditional Canny edge detection technology mainly uses customized equipment such as DSP and FPGA, but it has some problems, such as long development cycle, difficult debugging, resource consumption, and so on. At the same time, the adopted CUDA platform has the problem of poor cross-platform. In order to solve this problem, a fine-grained parallel Canny edge detection method is proposed, which is optimized from three aspects: task partition, vector memory access, and NDRange optimization, and CPU-GPU collaborative parallelism is realized. At the same time, the parallel Canny edge detection methods based on multi-core CPU and CUDA architecture are designed. The experimental results show that OpenCL accelerated Canny edge detection algorithm (OCL_Canny) achieves 20.68 times acceleration ratio compared with CPU serial algorithm at 7452 × 8024 image resolution. At the image resolution of 3500 × 3500, the OCL_Canny algorithm achieves 3.96 times the acceleration ratio compared with the CPU multi-threaded Canny parallel algorithm. At 1024 × 1024 image resolution, the OCL_Canny algorithm achieves 1.21 times the acceleration ratio compared with the CUDA-based Canny parallel algorithm. The effectiveness and performance portability of the proposed Canny edge detection parallel algorithm are verified, and it provides a reference for the research of fast calculation of image big data.

## 1. Introduction

With the development of computer science, image processing technology has achieved fruitful research results in recent years and has been widely used in industrial, military, medical, and other fields. As the most basic feature of the image, the edge feature of the image can greatly reduce the image information to be processed on the premise of retaining the shape information of the object [1]. The edge of a digital image contains a variety of useful information, which can be used to detect and recognize images. Digital image edge detection technology is widely used in image segmentation, motion detection, target tracking, face recognition, and other fields. Therefore, edge detection is one of the most important key technologies in the field of image processing [2].

At present, image edge detection algorithms mainly include edge detection algorithms based on wavelet transform, edge detection algorithms based on morphology, edge detection algorithms based on machine learning, and traditional edge detection algorithms [3]. The edge detection algorithm based on wavelet transform is used to transform the image with different scales. When the scale is small, the edge detail information is rich, and the positioning accuracy is high, but the anti-disturbance ability is poor. When the scale is large, the positioning accuracy is low and the anti-jamming ability is good, so it fuses the results of edge images of each scale, taking into account the positioning accuracy and anti-jamming ability to a certain extent, but the algorithm complexity is high [4]. The edge detection algorithm based on morphology uses the continuous movement of structural elements in the image to analyze and process the image and extracts different image features by selecting different structural elements for opening and closing and other operations. This algorithm is easy to implement, and can effectively remove the salt and pepper noise, but its edge location accuracy is not good. Edge detection algorithm based on machine learning has become a new research direction in recent years. In particular, the deep features of the image are extracted automatically by deep learning, and a good edge effect is obtained. Its disadvantage is that it requires a large number of samples of training and learning, and the computational complexity is high [5].

The traditional edge detection algorithms include the Roberts operator, Prewitt operator, Sobel operator, and so on. These algorithms are simple and easy to implement, but their denoising ability is poor, crack edge recognition is incomplete, and pseudo edges are easy to occur. Compared with these algorithms, the Canny edge detection operator used in this paper has a strong denoising ability and high detection accuracy [6]. The Canny edge extraction method was first proposed by John F. Canny in 1986 [7]. The Canny edge detection method is based on finding the local maximum of the gradient amplitude of the image. It uses the first derivative of the Gaussian filter to calculate the gradient amplitude. It uses the double-domain value method to detect the strong and weak edges respectively, and only when the strong and weak edges are connected, the weak edges where the strong edges are discontinuous will be included in the detection results. As a result, the influence of noise on the detection results can be reduced, and the detection results can achieve a better balance between noise and edge detection. However, the Canny operator also has obvious shortcomings. Due to the calculation flow of Gaussian filtering, gradient amplitude and direction calculation, non-maximum suppression, and double threshold processing, the algorithm has high complexity and slow operation speed, which is contrary to the fast and accurate application principle in practical engineering, which greatly restricts the engineering practicability of the algorithm. In order to improve the computing speed of the Canny operator, it is a good choice to use graphics processing unit (GPU) to parallelize processing. GPU has multiple threads for fast computing of large data with low coupling and high parallelism. At the same time, the parallel computing of GPU is becoming more and more mature in recent years, and its friendly programming operation and people-friendly price also make it possible to use GPU parallel processing Canny operator [8, 9]. In order to use GPU parallel processing Canny operator, it is necessary to optimize and parallelize the processing process of the Canny operator, so as to meet the requirements of GPU parallel processing. Through the optimization and transformation of the Canny operator, the processing mode of running GPU+CPU reduces the edge detection time of a 1280 × 720 image to less than 10 ms, which greatly improves the execution efficiency of the algorithm and lays a foundation for practical industrial applications.

For the problem that it is difficult to have both effectiveness and performance portability, this paper re-evaluates and analyzes all the steps of Canny edge detection according to the architecture of GPU, so that the key hot steps run completely on GPU. Based on the architecture of open computing language (OpenCL), the parallel implementation of the Canny edge detection algorithm (OCL_Canny) is completed. By analyzing the conventional inefficient memory access mode of single work-item and single pixel and the deficiency of low utilization of GPU memory, the method of vectorized memory access is proposed, which improves resource utilization and computational efficiency. At the same time, the OCL_Canny parallel algorithm also has the advantages of real-time and performance portability.

Therefore, the main contributions of this paper are as follows: (1) implement the Canny edge detection algorithm OCL_Canny through heterogeneous computing. (2) The OMP_Canny and CUDA_Canny parallel algorithms under the mainstream parallel computing framework of OpenMP and compute unified device architecture (CUDA) compare the time-consuming and accelerated performance with the OCL_Canny algorithm. (3) The performance of OCL_Canny on a heterogeneous GPU platform is evaluated, and the portability of its performance is analyzed.

The rest of the paper is arranged as follows. In Section 2, we review the research results of the Canny edge detection parallel algorithm, the existing implementation of FPGA and DSP computing architecture, the existing computing methods on graphics hardware, and the Canny algorithm on Hadoop cluster system. Section 3 summarizes the basic principles of OpenCL architecture and describes the Canny edge detection algorithm and the parallelism analysis of Canny operators. Section 4 describes the parallel computing process, design, and optimization solution of the Canny operator under OpenCL architecture. Section 5 discusses the design of OMP_Canny and CUDA_Canny parallel algorithms. Section 6 gives the relevant experimental results and makes an empirical evaluation of the performance of the OCL_Canny operator. Section 7 is the conclusion.

## 2. Background and introduction of related research

At present, many researchers have researched the implementation of the Canny edge detection parallel algorithm. SHI Weizhong et al. [10] proposed an optimization algorithm of Canny edge detection based on FPGA, which improves the processing speed under the 512 × 512 gray-scale image resolution. Jin et al. [11] chose ZC706 as the development platform to accelerate the edge detection of Canny based on the SDSoC development environment and obtained 16.97 times the speedup under the 512 × 512 gray-scale image resolution. Keqiang et al. [12] developed a Canny operator on the TI DSP TMS320C6678 processor, which improves the operator speed at 800 × 600 gray-scale image resolution. Xiangjiao et al. [13] implemented a parallel Canny algorithm based on threading building block (TBB) tool and C++ language and achieved 3.673 times acceleration ratio on 22.89 M gray-scale image based on a quad-core CPU. Yue et al. [14] realized the Canny edge detection algorithm on GPU using OpenGL, and the real-time performance of the algorithm was satisfied under 256 × 256 gray-scale image resolution. Bin et al. [15] proposed a method to quickly implement the Canny operator based on GPU+CPU, which can accelerate up to 5.39 times at 1024 × 1024 gray-scale image resolution. Jin et al. [16] proposed a Canny edge detection algorithm under OpenCL architecture, which achieves 1.42 times speedup at 2048 × 1536 gray-scale image resolution without considering data transmission. Mochurad [17] proposed a parallel computing method of the Canny algorithm using CUDA technology. The performance of the algorithm for processing gray-scale images with image frames of 10240 × 10240 is improved by 68 times. Horvath et al. [18] implemented the Canny edge detection algorithm on the CUDA platform and the performance of processing gray-scale image with an image size of 1280 × 720 was improved by 101 times. Some scholars have studied the implementation of the Canny edge detection algorithm in Hadoop cluster architecture, which improved the performance of batch processing images [19, 20].

Some scholars have proposed an improved Canny image edge detection method, which can effectively detect the gray-scale image edge of 512 × 512 image size in real time on FPGA [21, 22]. Lee et al. [23] implemented a Canny edge detector suitable for advanced mobile vision applications on FPGA under the slight sacrifice of detection effect, which saves the system execution time under various gray-scale image resolutions such as 512 × 512. However, the method reduces the effect of edge detection. Suwen et al. [24] proposed an improved Canny edge detection algorithm based on the FPGA platform, which improves the ability of weak edge detection. Shengxiao et al. [25] proposed an improved algorithm for edge detection of the Canny operator based on the GPU platform, which achieves 64 times speedup at 512 × 512 gray-scale image resolution.

Fuqiang et al. [26] designed the line segment detector algorithm with low error rate by using the Canny edge detection algorithm implemented on FPGA, which has the advantages of high reliability and high speed. Sivakumar and Janakiraman [27] proposed a new ROI region segmentation method for MRI images by implementing enhanced Canny operators on FPGA. Hongye [28] realized the fingerprint acquisition system based on DSP by optimizing the Canny edge extraction operator, which makes the identification speed of the fingerprint wireless acquisition system faster. Rongbao et al. [29] designed a verticality recognition system based on DSP+FPGA using the improved Canny algorithm. The results show that the system has high detection speed, and high precision and meets real-time requirements. Hanjun and Zeng [30] combined the Gaussian mixture model with Canny edge detection to extract the target contour, which shortens the computing time on the CUDA platform and meets the real-time requirements of video analysis. Tengzhang et al. [31] proposed a method based on the multi-feature Canny edge detection algorithm and the joint probability data association algorithm for moving multi-ship detection and tracking by on-orbit satellite. This method can detect and track the target quickly and accurately on the embedded GPU development platform.

To sum up, people mainly study the performance of the Canny algorithm from three aspects. The first is to accelerate the Canny operator in parallel under the architecture of multi-core CPU, FPGA, DSP, GPU, and Hadoop clusters. But the parallel effect of CPU is not strong, the performance-to-price ratio is not high, and the computing power is weak. The cost of FPGA is high, the resources are expensive, and the debugging is difficult. CUDA technology can only be carried out on NVIDIA graphics cards, and its cross-platform and portability are poor. Especially in the case of large-scale computing resource equipment, it is impossible to transplant and reuse the code on heterogeneous platforms. The second is to improve the performance of the improved Canny operator by improving the Canny operator in some aspects, such as optimizing the calculation process. The third is to apply the Canny operator to a variety of practical applications to achieve the acceleration of the application system under the parallel computing architecture. In these research results, most of the research results use a single parallel technology to improve the algorithm, without comparison with other parallel computing models, it cannot get the best acceleration effect. Current computer systems generally contain a variety of processors, such as CPU, GPU, and other types of processors. How to make reasonable and full use of a variety of computing resources on heterogeneous computing platforms will become very important.

In this paper, the storage of GPU is designed and used reasonably by using OpenCL parallel acceleration technology to realize the high-speed computing of the Canny image edge detection algorithm. By taking the three memory access modes of image data access on GPU, namely, global memory, local memory, and constant memory, as a starting point, the parallel implementation of image Gaussian filtering and image gradient in these three kinds of memory is analyzed and designed, so that the two operations can be realized more efficiently on GPU. In the process of research, GPU is used to realize image Gaussian filtering, image gradient, non-maximum value suppression of gradient image, and determining image edge points in parallel. To obtain the fast extraction of image edge as the goal, the calculation methods of image Gaussian filtering and image gradient are optimized and improved. From the perspective of saving storage resources and being more in line with the parallel programming architecture of GPU, the computing is improved, such as improving the operation method of the image template and extended image to make it more suitable for the parallel implementation under GPU. At the same time, the construction of pixel vectorization calculation under GPU is applied to the calculation of image Gaussian filtering and image gradient, which verifies the effectiveness of the vectorization parallel computing method of image Gaussian filtering and image gradient calculation.

## 3. Software model of algorithm

### 3.1. Overview of OpenCL

OpenCL is used for a parallel computing platform, which establishes the writing standard of parallel systems. OpenCL has a relatively wide range of applications, providing computing support for CPU, GPU, FPGA, and other devices, and has become a programming standard in the field of heterogeneous systems. OpenCL provides developers with a common programming interface and a development model for the underlying hardware layout.

OpenCL heterogeneous parallel architecture consists of four parts: platform model, execution model, storage model, and programming model. The four models support each other when the OpenCL system is running, and each model has its own unique role.

(1) Platform model

**Fig 1:**
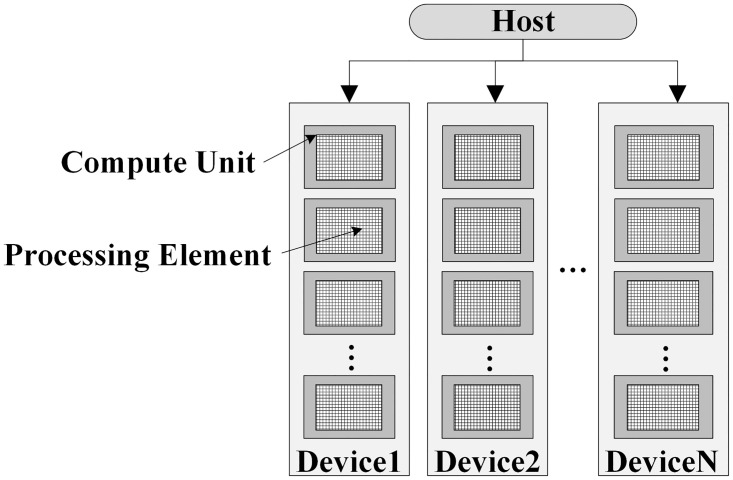
OpenCL platform model.

As shown in Fig 1, the OpenCL platform model consists of a Host connected to one or more OpenCL compute devices, which is used to realize the data exchange between the host and the OpenCL devices. CPU, GPU, and other processors that support OpenCL all belong to OpenCL devices. An OpenCL device can be divided into one or more compute units (CU), and each CU is composed of one or more processing elements (PEs) [32].

(2) Memory model

**Fig 2:**
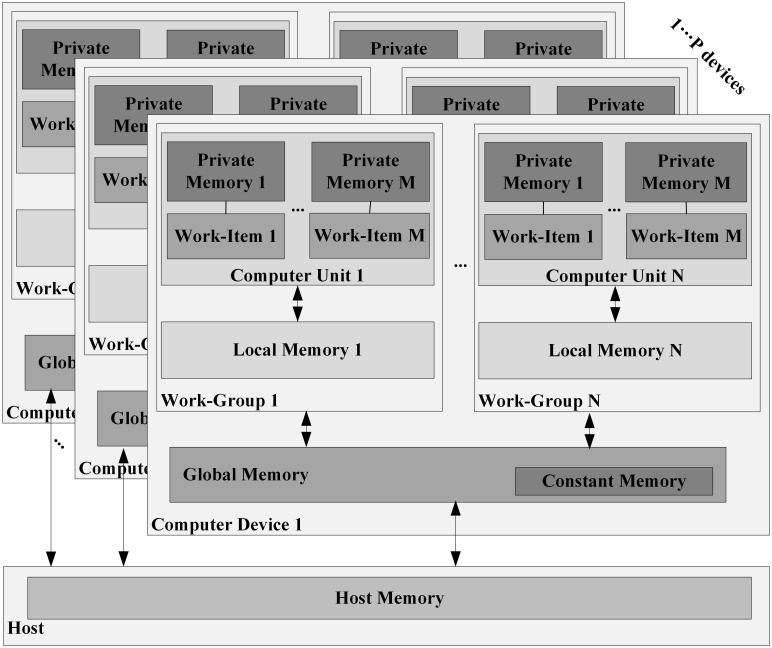
OpenCL memory model.

The memory in OpenCL architecture is divided into four different memory types. The location of each memory in the platform is shown in Fig 2. These four types of memory are global memory, constant memory, local memory, and private memory [33].

(3) Execution model

**Fig 3:**
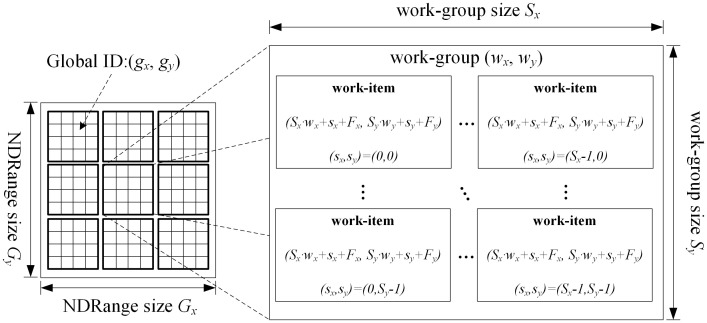
OpenCL execution model.

The execution model is shown in Fig 3. The execution model of OpenCL consists of two parts, one is the host system executing on the host machine, and the other is the kernel software executing on the OpenCL device. The OpenCL architecture manages the execution of kernel software in OpenCL devices by using context in the main system [34].

When the send kernel command is submitted on the host, the system plans an N-dimensional index space NDRang. The operation of each point in this space is called a work, which OpenCL calls a work-item. All work-items in the index space have their own unique coordinates, which serve as the global ID for each work-item. When sending kernel execution commands, the work-item is divided into several areas of the same size and becomes a collection of work-items, which are called work-groups. The number of work-items contained in all work-groups is the same, and similar to the global ID of work-items, work-groups also have ID, called work-group ID. Work-items in each work-group have a unique ID in the work-group, called a local ID. Fig 3 gives a two-dimensional index space, the size of the index space is *Gx* * *Gy*, in which a coordinate system is established to represent the global ID (*gx*, *gy*) of each work-item. The index space in the graph is divided into multiple work-groups with *Sx* * *Sy* work-items. OpenCL stipulates that *Gx* must be divisible by *Sx* and *Gy* must also be divisible by *Sy* [35].

(4) Programming model

OpenCL achieves the goal of acceleration by executing tasks in parallel, which is divided into task parallelism and data parallelism. Task parallel mode means that all the working nodes in the workspace of OpenCL devices are relatively independent, and the system can accelerate by executing multiple kernels at the same time or adding local kernel tasks to the kernel. Data parallel methods are commonly used, and multiple data are calculated in parallel so that the computational efficiency is significantly improved.

### 3.2. Algorithm theory

#### 3.2.1. Canny principle

The Canny operator fully reflects the mathematical characteristics of the optimal edge detector. It is the optimal approximation operator for the signal-to-noise ratio and location ability and is widely used in image processing and pattern recognition problems. The Canny operator not only has a good edge detection performance but also is insensitive to noise, even in a noisy environment, it also has a good edge detection effect. Therefore, the Canny operator can be applied to edge detection in different environments.

(1) Image preprocessing

The images to be detected are usually disturbed by noise. The amplitude of the gradient near the noise pixel is large, and the edge detection operator is easy to mistakenly detect the noise pixel as the edge pixel. Therefore, it is necessary to remove the noise in the image.

When the image is used for edge detection, the original data must be processed first. The input image is preprocessed by convolution filter with Gaussian filter to remove noise and reduce the influence of noise on gradient calculation, so as to better realize the effect of edge detection image segmentation. Therefore, image preprocessing requires convolution of the original image and Gaussian mask, and the processed image is more blurred than the original, which is conducive to image edge detection [36].

In the Canny operator, the smooth denoising of the image uses the first derivative of the 3 × 3 two-dimensional Gaussian function, and the Gaussian function and image convolution are shown in Eq (1).


$$\left\{\begin{array}{c} G\left(x,y,\sigma \right)=\frac{1}{2\pi \sigma^{2}}e^{-\frac{x^{2}+y^{2}}{2\sigma^{2}}}\\ H\left(x,y\right)=f\left(x,y\right)*G\left(x,y,\sigma \right) \end{array}\right.$$ (1)

In Eq (1), *f*(*x*, *y*) is the original image, *G*(*x*, *y*, *σ*) is the Gaussian function, *σ* is the standard deviation of the two-dimensional Gaussian function, and *H*(*x*, *y*) is the image smoothed by the Gaussian filter.

(2) Determine the amplitude and direction of the image gradient

**Fig 4:**
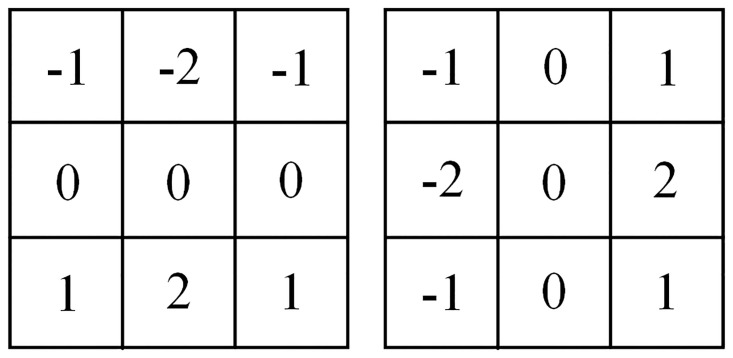
Sobel operator template.

The amplitude of the pixel gradient of the image *H*(*x*, *y*) can be calculated by the first partial derivative. In calculating the gradient direction, two 3 × 3 Sobel operators are used as the first order approximation of the partial derivatives in the *x* direction and *y* direction, as shown in Fig 4 [37].

Before determining the amplitude and direction of the image gradient, Eq (2) is used to solve the first order partial derivative matrix of the *x*-axis and *y*-axis direction.


$$\left\{\begin{array}{c} \begin{array}{l} P\left(x,y\right)=H\left(x+1,y-1\right)+2H\left(x+1,y\right)+H\left(x+1,y+1\right)\\ -H\left(x-1,y-1\right)-2H\left(x-1,y\right)-H\left(x-1,y+1\right) \end{array}\\ \begin{array}{l} Q\left(x,y\right)=H\left(x-1,y+1\right)+2H\left(x,y+1\right)+H\left(x+1,y+1\right)\\ -H\left(x-1,y-1\right)-2H\left(x,y-1\right)-H\left(x+1,y-1\right) \end{array} \end{array}\right.$$ (2)

The amplitude and direction of the gradient are calculated by the finite difference of the first order partial derivative. For the calculation results of the gradient amplitude, the non-maximum value suppression method is adopted. After processing, the gradient amplitude *M* and gradient direction *θ* at the pixel *H*(*x*, *y*) of the image can be calculated by Eqs (3) and (4) respectively [38].


$$M\left(x,y\right)=\sqrt{P{\left(x,y\right)}^{2}+Q{\left(x,y\right)}^{2}}$$ (3)

$$\theta \left(x,y\right)=\text{arctan}\left[\frac{Q\left(x,y\right)}{P\left(x,y\right)}\right]$$ (4)

(3) Perform non-maximum value suppression on the gradient amplitude image to determine the edge point

**Fig 5:**
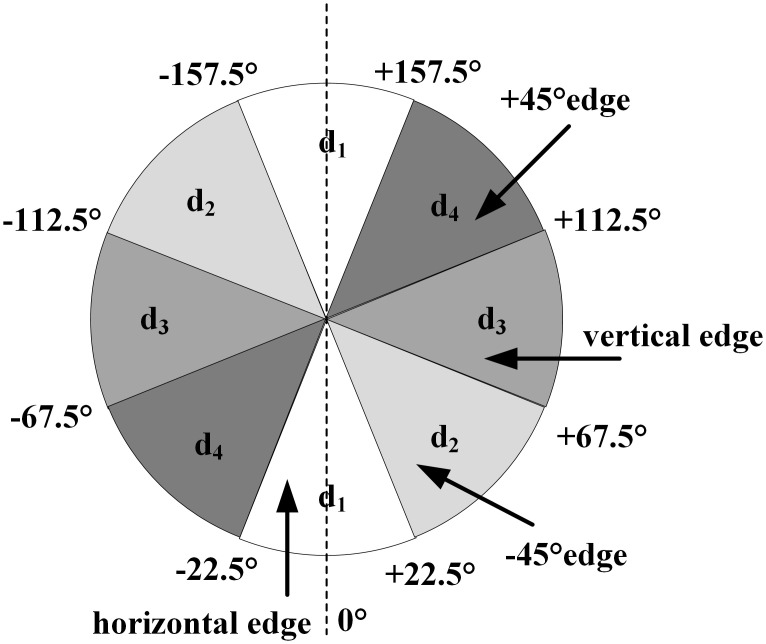
Sector chart.

Non-maximum value suppression is the key to find all the target edge points in the image. In order to determine the edge, it is necessary not only to get the global gradient but also to retain the maximum point of the local gradient and suppress the non-maximum value. In the 3 × 3 region, the edge can be divided into four directions: 0°, 45°, 90° and 135°. Similarly, the reverse direction of the gradient is also four directions (orthogonal to the edge direction). Therefore, in order to suppress the non-maximum value, all possible directions are quantized into four directions, as shown in Fig 5 [39].

In this way, the direction angle is regulated to the following four directions:

The vertical edge―gradient direction is horizontal: *θ*(*x*, *y*) ∈ [67.5°, 112.5°]⋃[−112.5°, −67.5°]
The 135° edge―gradient direction is 45°: *θ*(*x*, *y*) ∈ [22.5°, 67.5°]⋃[−157.5°, −112.5°)
The horizontal edge―gradient direction is vertical: *θ*(*x*, *y*) ∈ [0°, 22.5°]⋃[−22.5°, 0°]⋃(157.5°, 180°]⋃(−180°, −157.5°]
The 45° edge―gradient direction is 135°: *θ*(*x*, *y*) ∈ [112.5°, 1577.5°]⋃[−67.5°, −22.5°)

**Fig 6:**
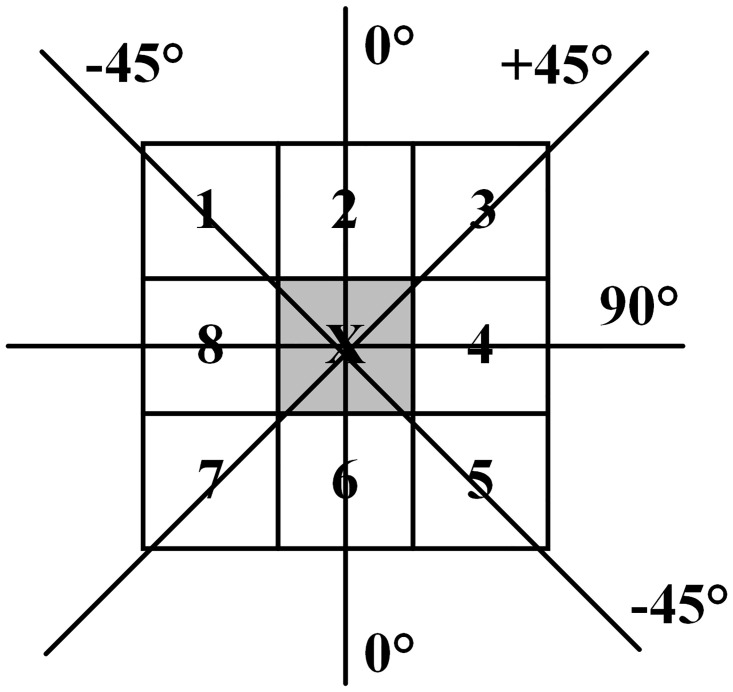
Pixel neighborhood structure.

In the 3 × 3 region, for each pixel in the image, there are only four possible directions connected to the adjacent points: 0°, 45°, 90°, and 135°, as shown in Fig 6 [40].

At the central pixel *H*(*x*, *y*) of each neighborhood is compared with two adjacent pixels along its corresponding gradient direction *θ*(*x*, *y*). If the gradient value *M*(*x*, *y*) at the center point is the largest, then the corresponding *f*(*x*, *y*) grayscale value is retained, otherwise, *f*(*x*, *y*) grayscale value is set to 0. As a result, the non-maximum value suppressed image *f*′(*x*, *y*) is obtained.

(4) Using double threshold algorithm to detect and connect edges of gradient images

In order to reduce the pseudo edge points, the double threshold algorithm is used to distinguish and connect the edges. If the edge strength is greater than the high threshold, it must be the edge point. If the edge strength is less than the low threshold, it must not be the edge point. If the edge intensity is greater than the low threshold and less than the high threshold, then see if there are any edge points in the adjacent pixels of this pixel that exceed the high threshold, if so, it is the edge point, if not, it is not the edge point.

Two thresholds, *T*_*L*_ and *T*_*H*_, are selected with a ratio of 1:2 or 1:3. For the image *f*′(*x*, *y*) processed by non-maximum value suppression processing, if the gradient value of the pixel is *M*(*x*, *y*) ≥ *T*_*H*_, then the pixel is marked as an edge pixel, namely, and the *f*(*x*, *y*) grayscale value is set to 255. If the gradient value of the pixel is *M*(*x*, *y*) ≤ *T*_*L*_, then the pixel is marked as a non-edge pixel, namely, and the *f*(*x*, *y*) grayscale value is set to 0. If the gradient value of the pixel is *T*_*L*_ < *M*(*x*, *y*) < *T*_*H*_, then the pixel is marked as "quasi-pixel", that is, and the *f*(*x*, *y*) grayscale value is set to 1. After the double threshold marking is completed, search for "quasi-pixel points" in the image, and select the positions of its 8 neighborhood points to find out whether there is a point with gradient value *M*(*i*, *j*) ≥ *T*_*H*_. If it exists, mark the pixel as an edge point, otherwise, mark the pixel as a non-edge pixel.

#### 3.2.2. Eliminate branches

**Fig 7:**
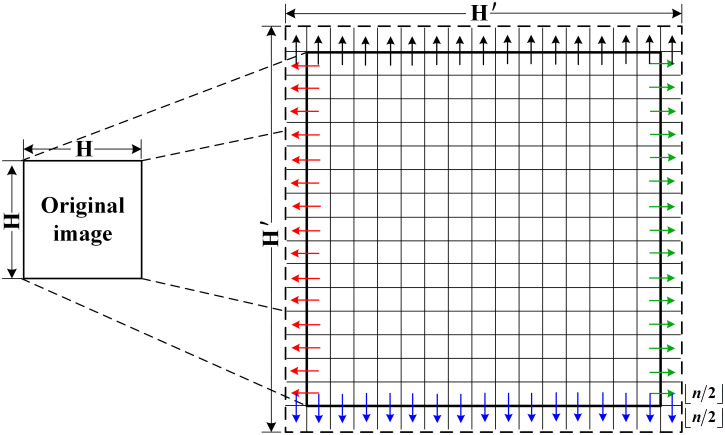
Boundary processing.

When using a template to traverse an image, the computation is out of bounds when traversing to the edge of the image. Therefore, the edge of the image to be processed is expanded before the calculation begins. The method of dealing with edge pixels in this paper is to make full use of the similarity of the image and take its own pixels to expand the original image. Suppose that the size of the original image is *H* × *H*, and the size of the image after edge expansion is *H*′ *H H*′, as shown in Fig 7, the solid line region and the dotted line region, respectively. When the neighborhood size is *n* × *n*, the edges of ⌊*n*/2⌋ pixels are filled around the original image. After extended preprocessing, there is no need for branch processing, which ensures a high degree of unity of the implementation process, and then improves the parallel potential of the algorithm.

In this paper, the method of even expansion is used to expand the edge of the original image. First of all, the gray values of all the pixels of the original image are filled into the middle part of the expanded edge image in turn. Then, fill the left boundary data of the original image into the corresponding left expansion area of the flared image, as pointed by the red arrow in Fig 7. Fill the right boundary data of the original image into the corresponding right expansion area of the flanged image, as pointed by the green arrow in Fig 7. Finally, fill the upper boundary data of the expanded image into the corresponding upper expansion area (including corners) of the final edge image, as pointed by the black arrow in Fig 7, and fill the lower boundary data of the expanded image into the corresponding lower expansion area (including corners) of the final edge image, as pointed by the blue arrow in Fig 7.

### 3.3. Serial system analysis

The 1024 × 1024 image size was used to test, the data bit depth was 8 bits, and the data format was BMP. When the CPU is Intel Core i7-8700K and the filter neighborhood size is 3 × 3, the time-consuming of each calculation step on the CPU is shown in Table 1. It can be seen from Table 1 that the most time-consuming step of the whole algorithm is the calculation of Canny edge detection, which includes the Gaussian filtering process for noisy images. The Canny edge detection step accounts for about 79.72% of the processing time of the whole Canny system. Therefore, the parallel acceleration in this paper will mainly focus on the Canny edge detection part. This conclusion is also applicable to the Canny edge detection system using images with different image contents and different image resolutions.

**Table 1:** Time-consuming of each module in the Canny algorithm.

Algorithm steps	Time-consuming by CPU (ms)	Occupancy time ratio (%)
Read in source image data	2.23	1.20
Extended source image	3.65	1.97
Gaussian template calculation	10.24	5.53
Initialize non-maximum value suppressed image	19.45	10.50
Canny edge detection	147.69	79.72
Output image edge extraction result	2.01	1.08
Total	185.27	100.00

In the calculation process of Canny edge detection, firstly, Gaussian filtering needs to take a filter window around the calculation point, and convolution calculation is carried out in this window. Then, the amplitude and direction of the image gradient need to be determined by using the Sobel operator, and then the gradient amplitude image is suppressed by non-maximum value, thus the non-maximum value suppression image is obtained. Finally, the double threshold algorithm is used to distinguish and connect the edges. Each pixel in the image data is processed in turn. When the image scale is large, the system will produce a large amount of computation. Therefore, reducing the computing time of Canny edge detection processing is one of the problems to be solved in this algorithm.

Suppose, the image size is *H* × *H* and the neighborhood size is *n* × *n*. Then

Process 1: The time complexity of the process of initializing a non-maximum value suppression image is *O*(*H*^2^).
Process 2: The time complexity of the step of expanding the edge of the image is *O*(*H*^2^) + *O*(*H* × *n*).
Process 3: The time complexity of the image Gaussian filtering step is *O*(*H*^2^*n*^2^).
Process 4: The time complexity of the process of determining the amplitude and direction of the image gradient is *O*(*H*^2^*n*^2^).
Process 5: The time complexity of non-maximum value suppression of gradient amplitude image is *O*(*H*^2^).
Process 6: The time complexity of the process of detecting and connecting edges of gradient images is *O*(9*H*^2^).

Therefore, the total time complexity of the Canny edge detection algorithm is: 2*O*(*H*^2^*n*^2^) + 3*O*(*H*^2^) + (*H* × *n*) + *O*(9*H*^2^). From the above analysis, it can be seen that process 3 ~ 6 is a functional part of the Canny edge detection algorithm with relatively high time complexity. Therefore, this paper should mainly focus on the parallel optimization of process 3 ~ 6, that is, the stage of Canny edge extraction. To sum up, the time complexity of the Canny edge detection algorithm is *O*(*H*^2^*n*^2^).

### 3.4. Algorithm parallel analysis

The parallelism analysis of the hot step process 3―process 6 in the Canny edge detection algorithm is carried out, and the time complexity of the algorithm is analyzed.

(1) Process 3: From the point of view of the image Gaussian filtering process, the *n* × *n* point multiplication is mainly carried out through the image pixel matrix and the Gaussian template matrix. The bottom layer of the algorithm processes a large amount of data, but the operation process is relatively simple. All pixels in the image can perform the same operation, there is no data dependence between each point of the target matrix, these operations can be performed in parallel, and the algorithm is a memory-intensive algorithm. In view of this, this paper realizes the optimization of the algorithm by improving the memory access efficiency and making rational use of GPU hardware resources.
(2) Process 4: The calculation of the amplitude and direction of the image gradient is to convolution each pixel with the Sobel operator in the *x* direction and *y* direction respectively, and then calculate the amplitude and direction of the gradient for the pixel. These computing processes are independent of each other and can be calculated in parallel.
(3) Process 5: Each central pixel is compared with two adjacent pixels in the same gradient direction to suppress non-maximum value pixels. The comparison process of each group is only related to the amplitude data of the current comparison pixels, but has nothing to do with other pixels. Each group of comparison processes can correspond to a work-item, so that process 5 can be executed in parallel.
(4) Process 6: The process of judging the edge points of pixels by using double thresholds does not affect each other and is independent of each other. It is beneficial to give full play to the performance advantages of GPU devices.

To sum up, the hot steps of the Canny edge detection algorithm, process 3―process 6, can be executed in parallel, which is suitable for implementation on GPU. Therefore, each pixel is assigned a processing element (PE) to process the corresponding pixel. Because all PEs perform the same computing process at the same time, the time complexity of the Canny edge detection parallel algorithm will be reduced to *O*(*n*^2^), which is a very small level of complexity. If all pixels are not processed in one kernel function, each PE will perform the Canny edge detection kernel function at least *H*^2^/*csum* times, where *csum* is the number of PE included in the GPU. In this case, the time complexity of the Canny edge detection parallel algorithm will be *O*(*H*^2^*n*^2^/*csum*). It should be noted that due to the large number of PE included in GPU, *csum* is always a large value. Therefore, there exists the time complexity of the Canny parallel algorithm *O*(*H*^2^*n*^2^/*csum*) ≪ *O*(*H*^2^*n*^2^).

## 4. OpenCL implementation of Canny edge detection algorithm

### 4.1. Parallel algorithm description

In order to maximize the effective use of GPU hardware multi-work-item resources, the reconstruction algorithm must strictly follow the OpenCL multi-work-item framework processing concept. When designing the kernel of image Gaussian blur, the kernel of calculating the amplitude and direction of image gradient, the kernel of suppressing non-maximum pixels, and the kernel of judging the edge points of pixels by GPU, the important foundation is that there is no correlation between pixel-by-pixel calculation. That is, the processing of each pixel is not related to each other. According to this feature, the Canny edge detection task can be divided into four kernels using GPU. The main function part of the Canny edge extraction parallel algorithm is described as follows.

1. **Algorithm 1 Canny edge detection parallel algorithm on OpenCL**

2. **Input**: Noisy image matrix *srcImageData* with image size *H* × *H*, array *GassTemplate*[0: *n* × *n* − 1] of the Gaussian convolution kernel, array *SobelTemplate*[0: *n* × *n* − 1] of the Sobel convolution kernel, each work-item is responsible for Gaussian filtering and processing of Sobel convolution in two directions of *BX* × *BY* pixels.

3. **Output**: Image matrix *desImageData* with canny edge detection

4. *srcImageData* ← input image with an image size *H* × *H*

5. *srcImageDataEx*← extended original image

6. *GassTemplate*[0: *n* × *n* − 1] ← calculate the Gaussian filter template

In the first kernel, the input image is blurred by Gaussian filter to suppress image noise. In order to improve the operation efficiency, the vectorization processing mode in which a single work-item is responsible for processing *BX* × *BY* pixels is adopted. The OpenCL kernel pseudocode that executes Gaussian blur is described below.

1. Initialize the global index *gx*, *gy* of the work-item in the *x* and *y* directions, respectively

2. Initialize the local index *lx*, *ly* of the work-item in the *x* and *y* directions, respectively

3. **for all** work-group in NDRange **par-do**

4. Load the input sub-image data that a work-group need to access from the global memory into a local memory of size *SubImage_ds*

5. **end for**

6. **for all** work-items in work-group **par-do**

7.  **for** *i* **=** 0 **to** *BX* **do**

8.   **for** *j* **=** 0 **to** *BY* **do**

9.    **for** *f*_*x*_ **=** 0 **to** *n* − 1 **do**

10.     **f for** *f*_*x*_ **=** 0 **to** *n* − 1 **do**

11.      *gaussPixel*[*i + j* * *BX*] ← Each work-item in the work-group does the convolution operation result of the corresponding pixel and the Gaussian template

12.     **end for**

13.    **end for**

14.    Output *gaussPixel*[*i + j* * *BX*]

15.   **end for**

16.  **end for**

**17. end for**

In the second kernel, the Sobel operation is performed on the Gaussian blur image to generate an image with highlighted edges. In order to improve the operation efficiency, the vectorization processing mode is also used to obtain the amplitude and direction of the image gradient. The OpenCL kernel pseudocode that performs Sobel filtering is described below.

1. **for all** work-group in NDRange **par-do**

2. Load the Gaussian filtering sub-image data that a work-group need to access from the global memory into a local memory of size *SubImage_ds*

3. **end for**

4. **for all** work-items in work-group **par-do**

5.  **for** *i* **=** 0 **to** *BX* **do**

6.   **for** *j* **=** 0 **to** *BY***do**

7.    **for** *f*_*x*_ = 0 **to** *n* − 1 **do**

8.     **for** *f*_*y*_ = 0 **to** *n* − 1 **do**

9.      *convolution*[*i*, *j* * *BX*] ← Each work-item in the work-group does the convolution operation result of the corresponding Gaussian filtering image pixel and the Sobel template

10.     **end for**

11.    **end for**

12.    Calculate the gradient amplitude and direction of pixels

13.   **end for**

14.  **end for**

15. **end for**

In the third kernel, non-maximum suppression is performed on Sobel results. The OpenCL kernel pseudocode that performs non-maximum suppression is described below.

1. **for all** work-items in NDRange **par-do**

2.  Judge whether the gradient amplitude of the neighborhood center pixel is the largest in the gradient direction

3. **end for**

In the fourth kernel, edge detection, and edge connection are performed on the gradient image. The OpenCL kernel pseudocode that executes marking edge points is described below.

1. **for all** work-items in NDRange **par-do**

**2**.  Using double threshold to judge whether the pixel of the gradient image is an edge point or not

3. **end for**

**4.** Transfer Canny edge detection results *desImageData* from global memory to host memory

### 4.2. Calculation process

**Fig 8:**
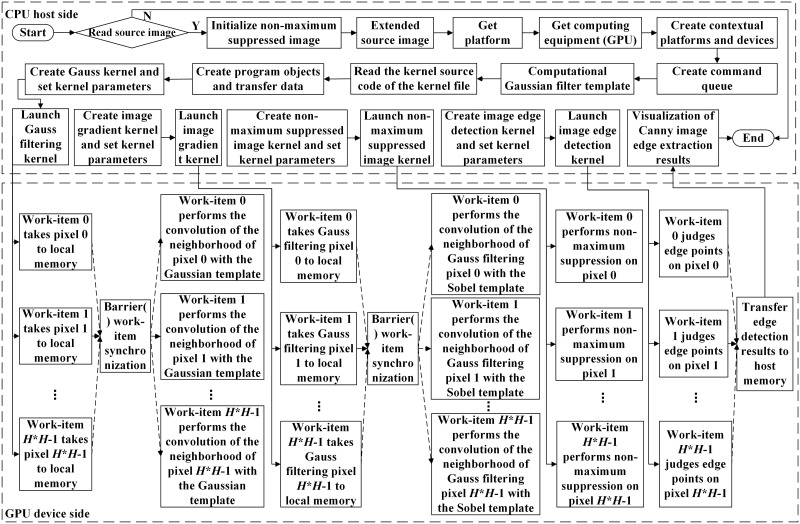
The OCL_Canny algorithm flow.

The edge detection process of the OCL_Canny parallel algorithm is shown in Fig 8.

The first step of the OCL_Canny parallel algorithm is to read the original image file to obtain image information and to expand the original image according to the size of the neighborhood window. Initialize the edge point image for subsequent calculation. Next, determine the platform for OpenCL execution, and then determine the device that performs the OpenCL calculation after determining the platform for execution. Create a context after determining the device.

After creating the context, you need to create a command queue. The operations such as extending the original image data transmission, Gaussian template data transmission, initializing the edge point image, and executing the kernel between the host and OpenCL devices are all done by queuing up to the command queue, and then the command queue passes each command to the OpenCL hardware unit for execution.

After that, the kernel code is compiled. First of all, the kernel source code is obtained from the host side and the program object is created, then the OpenCL device compiles and constructs the program object using the kernel source code, and finally constructs the kernel object to complete the compilation of the kernel code.

When the kernel function needs input parameters to provide calculation data, the corresponding application program interface function is called on the host side to complete the initialization of the input parameters. In addition, the work-group and work-item parameters used for execution on the device also need to be set in advance.

After the above operations are completed, the queuing operation is carried out, and the kernel function is sent to the corresponding command queue through the queuing command. The computing device interacts with the command queue and executes the corresponding kernel functions. The kernel functions of the OCL_Canny parallel algorithm include generating Gaussian filtered image kernel, generating gradient image kernel, generating edge point image kernel, and generating edge image kernel.

The operation of the kernel function is mainly the calculation and update of the incoming parameter variable, and the next call is the update status of the variable, and the four kernels are executed serially through CPU control. The execution process of the corresponding kernel function in this paper is as follows:

① Gaussian filtered image kernel. According to Eq (1), the extended image data is convoluted with the Gaussian template data and the information is updated.
② Gradient image kernel. According to Eqs (2) ~ (4), the Gaussian smoothing image data is convoluted with the Sobel template data, and the gradient amplitude and direction of the corresponding pixels are calculated.
③ Edge point image kernel. The gradient image is suppressed by non-maximum value, and the edge points of the image are preliminarily determined.
④ Edge image kernel. The edge points of the image are finally determined and connected by the double threshold method.

After the OpenCL device performs the calculation, it transmits the results of Canny edge detection back to the host side and destroys the allocated resources.

### 4.3. Acceleration strategy of the algorithm

The Canny edge detection algorithm has obvious data computing parallelism. The processing of Gaussian filtering, calculating the gradient of the image, suppressing non-maximum value pixels, and judging edge points with double thresholds are only related to the position of the image pixels, and the calculation process of each pixel is exactly the same.

**Fig 9:**
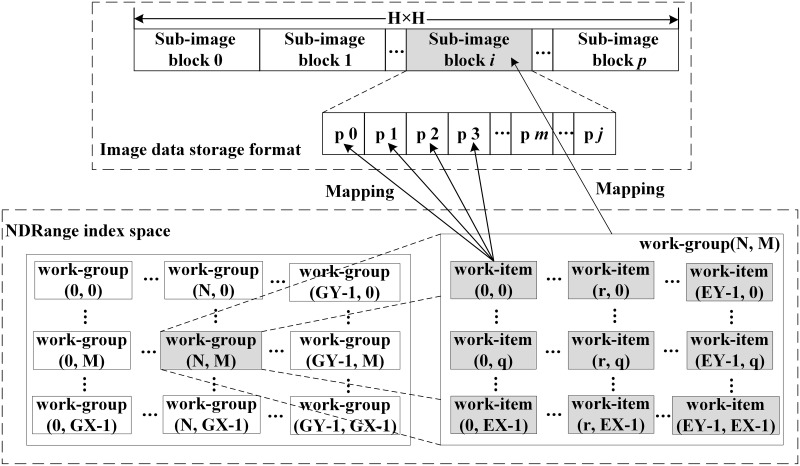
Corresponding relation of the work-item index and image pixel coordinate.

The mapping between the pixel and the OpenCL core mainly lies in the one-to-one logical correspondence between the work-item and the pixel. Fig 9 shows the mapping relationship between the NDRange workspace of the GPU and the image data matrix. The image frame *H* × *H* image data is arranged according to the one-dimensional linear organization in the system and can be decomposed into several non-overlapping sub-image blocks. Each sub-image block contains some pixels of the image. The kernel function creates an NDRange workspace that identifies the index, as shown in the lower dotted frame in Fig 9. Through the mapping of OpenCL work-items to image pixels, each OpenCL work-item uses a unique work-item index to calculate the data that needs to be processed to achieve maximum parallelism.

Processing more data in a shorter time has always been one of the goals of high-performance computing. OCL_Canny parallel algorithm proposes a vectorization method to process multiple pixels at a time for each work-item. In the algorithm, the Gaussian filtering operation and the Sobel image gradient operation of four adjacent pixels in the sub-image block are scheduled on one work-item in turn. The calculation of the four output results is completed on the same work-item, and each cycle can complete the calculation of the output result of one pixel, thus completing the traversal of the four pixels. The coordinate transformation of the pixel is shown in Eq (5).


$$\begin{array}{l} lx=get\_local\_id\left(0\right),ly=get\_local\_id\left(1\right)\\ gx=get\_global\_id\left(0\right),gy=get\_global\_id\left(1\right) \end{array}$$ (5)

Among them, *lx*, *ly* represents the local ID of the work-item in the *x*, *y* direction respectively in the work-group. *gx*, *gy* represents the global ID of work-items in the *x*, *y* direction respectively in the workspace. Through the four variables, the precise scheduling of OpenCL work-items can be completed.

### 4.4. Algorithm optimization

#### 4.4.1. Data storage adjustment

(1) Local memory optimization

In the processing of the four tasks of the Canny operator, the calculation of the boundary points in the work-group needs to cross the boundary. In order to prevent the image from crossing the boundary, the original image is extended to an expanded image in this paper. Suppose the template size is *n* × *n* and the original image size is *H* × *H*. When calculating the Gaussian filter, gradient amplitude, and direction, the size of the expanded image is (*H* + *n* − 1) × (*H* + *n* − 1). In this case, the basic data area size of non-maximum value suppression and double threshold judgment of gradient image is *H* × *H*, then the size of the extended data area is also(*H* + *n* − 1) × (*H* + *n* − 1).

Because the local memory is located on the GPU, the local memory has higher bandwidth and lower latency than the global memory, and the access speed of the local memory is much higher than that of the global memory. In the OCL_Canny parallel algorithm with global memory, it is necessary to access the global memory (*H* + *n* − 1)^2^ × *n*^2^ × 4 times. By fetching the extended data area data from the global memory to the local memory, and taking the local memory as the memory for accessing the data when the work-item is calculated, the number of visits to the global memory is reduced to (*H* + *n* − 1)^2^ × 2*n* × 4 times. Therefore, the optimized OCL_Canny parallel algorithm can significantly reduce the number of times of accessing global memory and greatly improve the access efficiency of the GPU.

(2) Constant memory optimization

In the OCL_Canny parallel algorithm, image preprocessing and image gradient calculation are completed under convolution computation. Since the convolution operation of the image needs to traverse the image pixels, when processing each pixel, it is necessary to read the corresponding pixels in the *n*^2^ neighborhood to multiply and add with the Gaussian template and the Sobel template. It requires frequent access to memory and the calculation is very time-consuming. Considering that the constant memory has a cache mechanism when the access is hit, there is only one clock cycle delay. Therefore, in order to improve the access efficiency, the Gaussian template and the Sobel template are stored in the constant memory for all work-items to read. The constant memory has a 64 KB cache, and the storage space needed to store the Gaussian template and the Sobel template is (3*n*^2^ × 4) B, which meets the maximum space requirements of the constant memory of 64 KB.

(3) Data reusability

From the processing flow of the Canny operator, we can see that each step of the algorithm is designed with a kernel, which is implemented with four kernels. Because there is a logical correlation between the kernel, that is, the results after the execution of the previous task need to be provided to the next task. Therefore, each kernel can store the calculation results in the global memory and wait for the next kernel to be read. By improving the data reusability, the data transmission times between CPU and GPU are reduced, thus the memory communication delay is hidden.

#### 4.4.2. NDRange optimization

According to different GPU hardware, change the number of work-items in each work-group in the kernel function to achieve optimal performance. If the number of work-items is too small, it will cause most of the PEs to be idle, waste resources, and low performance. If the number of work-items is too large, due to the limitation of hardware resources, it may not be possible to actually start enough active work-items, which will cause too many work-items to be in a blocked state and also cause performance degradation. Therefore, in order to ensure the optimal performance of the OCL_Canny parallel algorithm on the GeForce GTX 1050 graphics card, the operation time of the algorithm is measured under different work-group dimensions, and the specific test data are shown in Table 2.

**Table 2:** Operation time of the OCL_Canny parallel algorithm.

Image size	Parallel time corresponding to different work-group sizes (ms)				
	4×4	8×8	16×16	24×24	32×32
256×256	3.87	3.62	3.02	3.58	4.27
512×512	6.75	5.09	4.28	5.01	5.39
1024×1024	20.78	12.78	10.31	12.66	13.42

It can be seen from the above experimental results that for GeForce GTX 1050 graphics cards, the maximum number of work-items per work-group is 1024. An error will be reported when running over this number. At the same time, 16 × 16 is also the best operating efficiency point.

## 5. Other parallel schemes

### 5.1. The OMP_Canny parallel algorithm

The parallel processing of the Canny edge detection algorithm is realized by using OpenMP parallel technology. With the addition of parallel task scheduling at the top level, this coarse-grained parallel processing method can realize Gaussian filtering, calculating image gradient, suppression of non-maximum value pixels, and parallel computing of edge points judged by double thresholds. This paper mainly adopts the static scheduling mode, and the specific parallel process: when *m* CPU cores are allocated to process the image size *H* × *H*, each core (or thread) will process (*H* × *H*)/*m* image data.

The parallel model of OpenMP is in the form of Fork-Join, and the area between Fork and Join is a parallel region. When the original thread encounters a parallel structure instruction, it creates a thread group and executes the next instruction in parallel, that is, the Fork action. When exiting the parallel structure, only the original thread continues to execute, and the other threads end, that is, the Join action. The OMP_Canny parallel algorithm executes the Fork action to open the parallel region at the starting position of the Gaussian filtering operation, and executes the Join action to end the parallel region when the detection of all edge points of the image is completed, thus forming the following four parallel regions.

(1) Parallel region of smooth image. First, initialize the variable, then convolution the neighborhood of the pixel with the Gaussian filter template, and finally, update the convolution value back to the corresponding position of the image.
(2) The parallel region that determines the amplitude and direction of the image gradient. Firstly, the variables are initialized in *x*, *y* direction, and then the neighborhood of the pixel after the Gaussian filter is convoluted with the Sobel filter template in *x*, *y* direction, respectively. Finally, the gradient amplitude and gradient direction of the pixel are calculated.
(3) Determine the parallel region of the non-maximum value suppressed gradient image. According to the gradient direction of the pixel, the pixel is suppressed by non-maximum value, and the non-maximum value suppression image is obtained.
(4) Determine the parallel region of image edge points. Using a double threshold algorithm to detect edge points and connect the edges of non-maximum value suppressed images.

### 5.2. The CUDA_Canny parallel algorithm

According to the parallelism analysis of the Canny algorithm, there are obvious data computational parallelism in image Gaussian filtering, calculating the amplitude and direction of image gradient, non-maximum value suppression gradient image generation, and image edge point detection. The mapping between the image and the execution thread mainly lies in the correspondence between the pixel and the CUDA thread. If the image size is *H* × *H* and the GPU has *a* streaming multiprocessors, the image data of *H* × *H* size is inputted into the GPU memory. In the software architecture, each GPU streaming multiprocessor contains *b* thread blocks and each thread block contains *c* threads, so it can be calculated that each thread can complete the Gaussian filtering processing of (*H* × *H*)/(*a*×*b*×*c*) pixels. The parallel processing of the gradient amplitude and gradient direction of each pixel is the same. In the experiment of CUDA_Canny parallel algorithm implementation, the GPU used is GTX 1050 with 24 streaming multiprocessors, each streaming multiprocessor contains 32 thread blocks, and each thread block has 1024 threads.

## 6. Data testing and result discussion

### 6.1. Experimental conditions

(1) The hardware platform is built. This experimental scheme uses two different environments with heterogeneous computing capabilities, and the specific hardware configuration information is shown in Table 3.
(2) The software platform is built. The operating system is Microsoft Window 10 64-bit, the GPU application programming interface is CUDA 10.2, the OpenCL version is AMD APP SDK 3.0, and the development environment is Microsoft Visual Studio 2017.

**Table 3:** Performance parameters of GPU Computing platform.

Configuration number	CPU type	CPU frequency	Memory/GB	GPU type	Video memory	Number of GPU cores	Number of SM	Number of blocks per SM	Number of threads per block
Configuration 1	Intel Core i7-8700K (six cores)	3.7 GHz	4	Geforce GTX 1050	3 GB GDDR5	768	24	32	1024
Configuration 2	AMD Ryzen 5 3600XT (six cores)	3.8 GHz	4	Radeon RX 560	4 GB GDDR5	896	28	32	1024

### 6.2. Image quality evaluation

#### 6.2.1. Visual effect comparison

**Fig 10:**
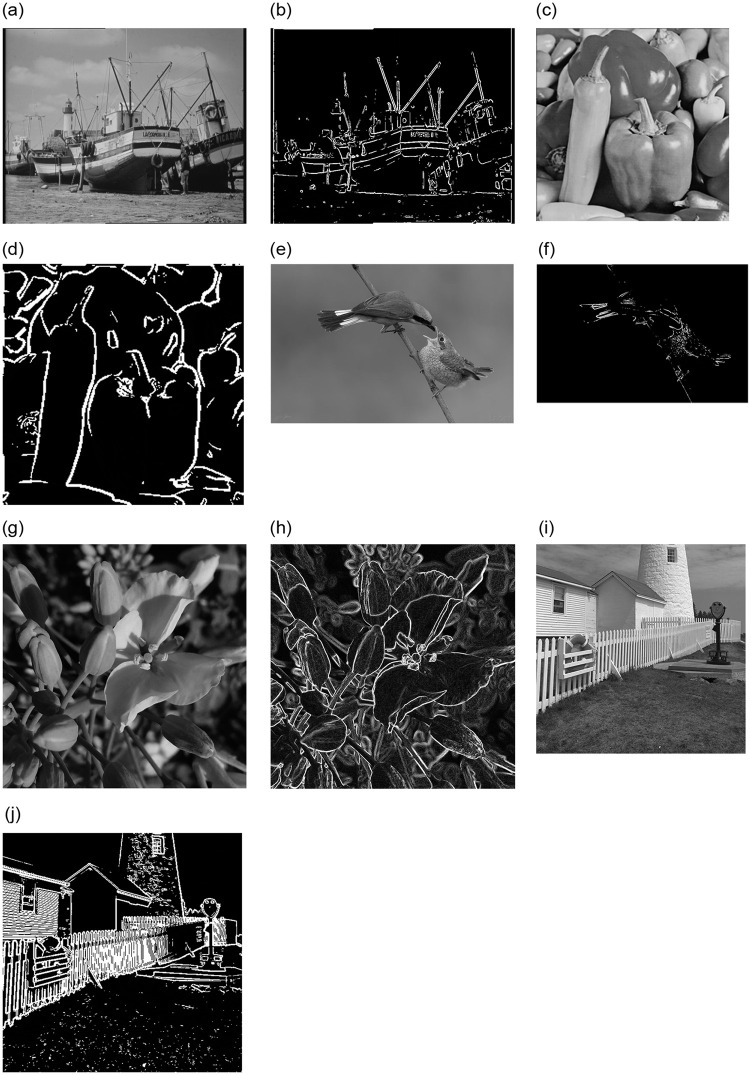
Edge detection effect images of four different Canny algorithms.

In order to verify the effectiveness of this method, five images are selected as test objects. The resolutions of the images "Boats", "Peppers", "Bird", "Flower" and "Light-house" are 720×576, 256 × 256, 990×650, 1024×1024 and 512 × 512, respectively. Four serial/parallel Canny edge detection algorithms are tested and the experimental results are shown in Fig 10.

As can be seen from Fig 10, the Boats result image is basically connected and undisconnected, with good coherence and high definition. The outline lines of many kinds of chili peppers in the Peppers image are basically smooth. The outline lines of the beak and tail feathers in the Bird image are very clear and smooth, with almost no breakpoints. The outline of the petals in the Flower image is closed and can be seen clearly. The texture of the exterior wall, the fence, and the edges of the eaves are very clear in the Light-house result image.

**Table:** 

Boats					
Peppers					
Bird					
Flower					
Lighthouse					
	(a) Original image	(b) CPU_Canny edge detection result	(c) OpenMP_Canny edge detection result	(d) CUDA_Canny edge detection result	(e) OpenCL_Canny edge detection result

It can be seen from Fig 10 that the effects of the serial Canny algorithm and optimized accelerated algorithm are basically the same, and the four edge detection operators can obtain image edges more accurately. The above experiments show that the OCL_Canny parallel algorithm is feasible.

#### 6.2.2. Comparison of evaluation parameters

In order to evaluate the effect of image edge detection, the average gradient value of the image is selected as the evaluation parameter. The Average Gradient (AG) is also called image sharpness, which is an indicator of the rate of gray change in image. The average gradient is defined as:

$$D_{x}\left(i,j\right)=I\left(i,j\right)-I\left(i+1,j\right)$$ (6)

$$D_{y}\left(i,j\right)=I\left(i,j\right)-I\left(i,j+1\right)$$ (7)

$$AG=\frac{1}{M\times N}\sum_{i=0}^{M-1}\sum_{j=0}^{N-1}\sqrt{\frac{1}{2}\left(D_{x}^{2}\left(i,j\right)+D_{y}^{2}\left(i,j\right)\right)}$$ (8)

Among them, *D*_*x*_(*i*, *j*), *D*_*y*_(*i*, *j*) denotes the gradient of the image in the *x* direction and *y* direction respectively. *I*(*i*, *j*) is the gray value of the image (*i*, *j*), (*i*, *j*) is the position index of the pixel in the image, and the image frame size of the image *I* is *M* × *N*. The image average gradients of different Canny edge detection algorithms are shown in Table 4.

**Table 4:** The average gradient of the Canny edge detection algorithm in different images.

Image name	No processing	CPU_ Canny	OMP_ Canny	CUDA_ Canny	OCL_ Canny
Boats	6.64	13.23	13.23	13.23	13.23
Peppers	7.51	19.06	19.06	19.06	19.06
Bird	2.67	4.26	4.26	4.26	4.26
Flower	3.12	18.21	18.21	18.21	18.21
Lighthouse	13.08	30.64	30.64	30.64	30.64

As can be seen from Table 4, the average gradient of the OCL_Canny parallel algorithm on the test image set is larger than that of the original image, indicating that the algorithm in this paper is the best in preserving edge details. At the same time, the average gradient data of the test image under serial/parallel Canny edge extraction are the same. It shows that the OCL_Canny parallel algorithm is correct and feasible.

Standard deviation is the first-order and second-order statistical characteristics of the gray values of all pixels in an image, which mainly reflects the overall average brightness and contrast of the image. The specific definition is as follows:

$$\mu =\frac{1}{M\times N}\sum_{i=0}^{M-1}\sum_{j=0}^{N-1}I\left(i,j\right),\sigma =\sqrt{\frac{1}{M\times N}\sum_{i=0}^{M-1}\sum_{j=0}^{N-1}{\left(I\left(i,j\right)-\mu \right)}^{2}}$$ (9)

Where *μ* is the average gray-scale of the image and *σ* is the standard deviation of the image. The standard deviations of the processing results of different Canny edge detection algorithms are shown in Table 5.

**Table 5:** The standard deviation of the Canny edge detection algorithm in different images.

Image name	No processing	CPU_ Canny	OMP_ Canny	CUDA_ Canny	OCL_ Canny
Boats	49.19	63.14	63.14	63.14	63.14
Peppers	53.26	80.32	80.32	80.32	80.32
Bird	21.85	37.61	37.61	37.61	37.61
Flower	49.56	52.58	52.58	52.58	52.58
Lighthouse	53.68	101.34	101.34	101.34	101.34

As can be seen from Table 5, the standard deviation of the OCL_Canny parallel algorithm on the test image set is larger than that of the original image, indicating that the result processed by this algorithm has high contrast and more edge information is extracted. At the same time, the standard deviation data of the test image under serial/parallel Canny edge extraction are the same. It shows that the OCL_Canny parallel algorithm is correct and feasible.

### 6.3. Analysis of experimental data

#### 6.3.1. Operation time comparison

In order to verify the high performance of the proposed algorithm, nine groups of images of different sizes are selected for experimental analysis. CPU_Canny algorithm, OMP_Canny algorithm, and CUDA_Canny algorithm measured the execution time in the configuration 1 environment, while the OCL_Canny algorithm measured execution time in the configuration 1 and configuration 2 environment, respectively. After many times of execution, the average value of the system is taken as the execution time. The time-consuming statistics are shown in Table 6.

**Table 6:** Time-consuming comparison of Canny algorithms under different architectures.

Image resolution (px)	CPU_Canny (ms)	Parallel time (ms)			
		OMP_Canny	CUDA_Canny	OCL_Canny (AMD)	OCL_Canny (NVIDIA)
256×256	9.45	2.90	3.26	3.14	3.02
512×512	40.12	11.33	4.59	4.41	4.28
1280×720	103.26	27.46	11.02	10.24	9.87
1024×1024	147.69	35.58	12.49	10.68	10.31
1600×1200	309.26	67.67	21.11	19.42	18.34
2048×1536	548.43	112.85	31.83	29.05	28.74
3500×3500	2311.45	459.54	120.29	117.62	115.97
4828×4828	4024.03	762.13	207.96	204.61	199.80
7452×8024	10105.12	1867.84	516.74	513.15	488.64

**Fig 11:**
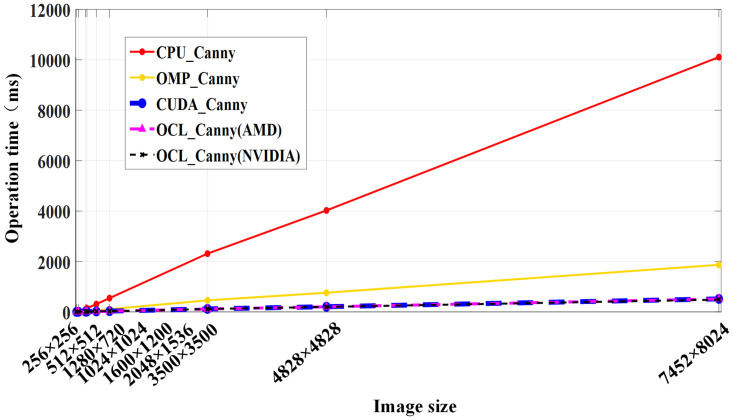
Time-consuming analysis of the Canny algorithm.

In order to more intuitively analyze the time characteristics of the Canny algorithm, it is shown in Fig 11. As can be seen from Fig 11, with the continuous increase of the size of nine groups of images, the time-consumption of the Canny algorithm under different computing architectures increases linearly. The time-consuming of the CPU_Canny serial algorithm is gradually approaching to *O*(*H*^2^*n*^2^). The experimental results are consistent with the theoretical analysis of time complexity. The time-consuming curve of the Canny algorithm under OpenMP architecture shows a steady upward trend of a small slope. On the other hand, the time-consuming curve of the Canny algorithm under CUDA and OpenCL architecture almost coincides with the horizontal axis in the graph, that is, the time-consuming change of the algorithm is very small with the increase of the amount of data processed. The time complexity of the Canny edge detection parallel algorithm *O*(*H*^2^*n*^2^/*tsum*) reflects that the time consumption of the parallel algorithm does not increase significantly with the increase of image resolution. The time-consuming trend of the OCL_Canny parallel algorithm in Fig 11 is basically consistent with the situation reflected in its time complexity.

Literature [15] and literature [17] reported the implementation results of the Canny algorithm under CUDA architecture, literature [16] reported the implementation results of the Canny algorithm under OpenCL architecture, and literature [11] reported the implementation results of the Canny algorithm under FPGA computing architecture. The data shown in these literatures are compared with the time-consuming of the algorithms in this paper, as shown in Table 7. According to Table 7, the Canny serial algorithm implemented in references [11, 15, 17] takes more time to run on the six groups of images involved than the CPU_Canny algorithm in this paper. The four parallel algorithms in literature [11], literature [15], literature [16], and literature [17] all consume significantly more time than the CUDA_Canny and OCL_Canny parallel algorithms in this paper on the 10 sets of images involved. Therefore, the OCL_Canny parallel algorithm has the advantage of time-consuming compared with other schemes.

**Table 7:** Comparison of operation time in related literature (1).

Image resolution (px)	CPU algorithm (ms)				CUDA algorithm (ms)			Literature [11]	OpenCL algorithm (ms)	
	Literature [11]	Literature [15]	Literature [17]	CPU_ Canny	Literature [15]	Literature [17]	CUDA_ Canny		Literature [16]	OCL_Canny (NVIDIA) (NVIDIA)
256×256	—	10.00	—	9.45	5.00	—	3.26	—	—	3.02
512×512	78.24	41.00	—	40.12	22.00	—	4.59	4.61	—	4.28
640×480	—	—	270.74	61.44	—	106.51	4.80	—	—	4.37
1280×720	—	—	—	103.26	—	—	11.02	—	19.04	9.87
1024×1024	—	149.00	—	147.69	82.00	—	12.49	—	—	10.31
1600×1200	—	—	—	309.26	—	—	21.11	—	39.46	18.34
2048×1536	—	—	—	548.43	—	—	31.83	—	58.03	28.74
4096×2160	—	—	7332.31	1513.40	—	405.64	70.52	—	—	67.86
3500×3500	—	—	—	2311.45	—	—	120.29	—	239.89	115.97
10240×10240	—	—	111740.55	19326.90	—	1643.23	813.97	—	—	810.25

In order to describe the performance of the OCL_Canny parallel algorithm more accurately, the computing time of three core kernel tasks in the OCL_Canny parallel algorithm is compared with the data in the literature [18]. Because the gray-scale image is input in the experiment, the performance comparison of the gray-scale kernel is no longer carried out. In addition, the computing time of the CPU_Canny algorithm is also compared with that of the serial algorithm in literature [18], so that the performance of the OCL_Canny parallel algorithm can be explained more accurately. The specific data are shown in Table 8.

**Table 8:** Comparison of operation time in related literature (2).

Image resolution (px)	CPU algorithm (ms)				GPU algorithm (ms)			
	640×480		1280×720		640×480		1280×720	
Comparison object	Literature [18]	CPU_ Canny	Literature [18]	CPU_ Canny	Literature [18]	OCL_Canny (NVIDIA)	Literature [18]	OCL_Canny (NVIDIA)
Gaussian Blur Kernel	—				0.04	0.03	0.12	0.11
Sobel Filter Kernel					0.33	0.11	0.94	0.29
Suppression Kernel					0.06	0.04	0.16	0.12
Total	54.73	33.10	166.19	99.01	0.51	1.37	1.35	3.07

According to Table 8, under two groups of test images, on the one hand, the computing time of the CPU_Canny serial algorithm is much shorter than that of serial implementation in literature [18]. The total time in Table 8 includes only the execution parts that are closely related to the three kernel tasks. The operation time in Table 7 includes not only the time in Table 8 but also the execution time of system initialization, dynamic generation of the Gaussian template, image edge expansion, and so on. The Suppression Kernel data for the OCL_Canny algorithm in Table 8 includes two execution tasks: non-maximum suppression and marking edge points. On the other hand, the operation time of the three kernels of the OCL_Canny parallel algorithm is shorter than that of the literature [18]. This shows that the parallel optimization effect of the most core computing task in the Canny algorithm is better. However, the total execution time of the OCL_Canny parallel algorithm is higher than that of the literature [18]. The main reasons are as follows: (1) the calculation range of the OCL_Canny parallel algorithm is the expanded edge image *H*′ × *H*′, not the original image *H* × *H*. (2) the total execution time of the OCL_Canny parallel algorithm includes the kernel computation time, data transmission time between host and device, storage space allocation time, and so on. However, the coverage of the total execution time in the literature [18] is not clear.

When the image resolution is 640 × 480, the ratio of computing time between the host and the GPU device is (4.37 − 1.37)/1.37 = 2.19. When the image resolution is 1280 × 720, the ratio of computing time between the host and the GPU device is (9.87 − 3.07)/3.07 = 2.21. This shows that the time consumed by the host in the OCL_Canny parallel algorithm is about twice as long as that of the GPU device.

#### 6.3.2. Accelerated performance analysis

(1) Speedup discussion

In order to select a high-performance Canny parallel algorithm, the speedup is used as the performance measure.

**Definition 1:** speedup *S*_*OMP*_ is defined as the time-consuming comparison between the CPU_Canny serial algorithm and the OMP_Canny parallel algorithm. The calculation equation of *S*_*OMP*_ is

$$S_{OMP}=\frac{T_{CPU\_Canny}}{T_{OMP\_Canny}}$$ (10)

**Definition 2:** speedup *S*_*CUDA*_ is defined as the time-consuming comparison between the CPU_Canny serial algorithm and the CUDA_Canny parallel algorithm. The calculation equation of *S*_*CUDA*_ is

$$S_{CUDA}=\frac{T_{CPU\_Canny}}{T_{CUDA\_Canny}}$$ (11)

**Definition 3:** speedup *S*_*OCL*_ is defined as the time-consuming comparison between the CPU_Canny serial algorithm and the OCL_Canny parallel algorithm on the corresponding GPU platform. The calculation equation of *S*_*OCL*_ is

$$S_{OCL}=\frac{T_{CPU\_Canny}}{T_{OCL\_Canny}}$$ (12)

**Definition 4:** relative speedup *RS*_*CMP-OCL*_ is defined as the time-consuming comparison between the OMP_Canny parallel algorithm and the NVIDIA GPU-based OCL_Canny parallel algorithm. The calculation equation of *RS*_*CMP-OCL*_ is

$$RS_{OMP-OCL}=\frac{T_{OMP\_Canny}}{T_{OCL\_Canny}}$$ (13)

**Definition 5:** relative speedup *RS*_*CUDA-OCL*_ is defined as the time-consuming comparison between the CUDA _ Canny parallel algorithm and the NVIDIA GPU-based OCL_Canny parallel algorithm. The calculation equation of *RS*_*CUDA-OCL*_ is

$$RS_{CUDA-OCL}=\frac{T_{CUDA\_Canny}}{T_{OCL\_Canny}}$$ (14)

The speedup achieved by the OMP_Canny, CUDA_Canny, and OCL_Canny parallel algorithms on each group of test images is shown in Table 9.

**Table 9:** Acceleration effect of the Canny algorithm on different platforms.

Image resolution (px)	Speedup				Relative speedup	
	*S*_*OMP*_	*S*_*CUDA*_	*S*_*OCL*_ (AMD)	*S*_*OCL*_ (NVIDIA)	*RS*_OMP-OCL_	*RS*_CUDA-OCL_
256×256	3.26	2.90	3.01	3.13	0.96	1.08
512×512	3.54	8.74	9.10	9.37	2.65	1.07
1280×720	3.76	9.37	10.08	10.46	2.78	1.12
1024×1024	4.15	11.82	13.83	14.32	3.45	1.21
1600×1200	4.57	14.65	15.92	16.86	3.69	1.15
2048×1536	4.86	17.23	18.88	19.08	3.93	1.11
3500×3500	5.03	19.22	19.65	19.93	3.96	1.04
4828×4828	5.28	19.35	19.67	20.14	3.81	1.04
7452×8024	5.41	19.56	19.69	20.68	3.82	1.06

**Fig 12:**
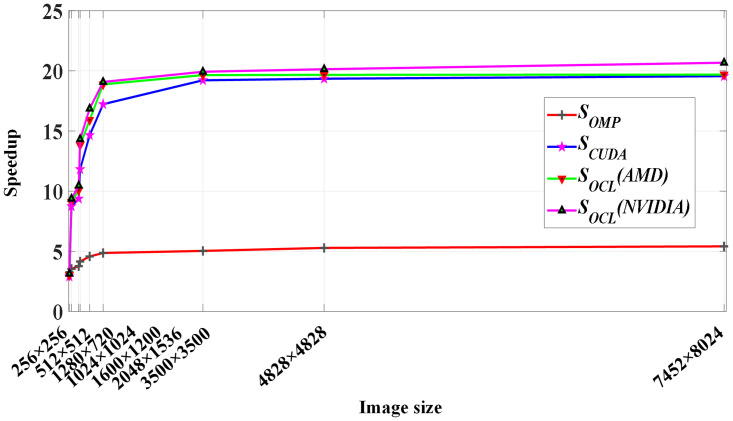
Performance acceleration of the Canny algorithm.

Fig 12 shows the speedup change of the Canny parallel algorithm under different image data sizes. Under different parallel computing architectures, the Canny algorithm achieves a certain speedup. With the increase of image resolution, *S*_*OMP*_ gradually becomes larger, indicating that the acceleration effect of the OMP_Canny parallel algorithm is more obvious when dealing with large images. When the image resolution is low, the acceleration effect of the CUDA_Canny and OCL_Canny parallel algorithms is not obvious. Because GPU computing needs to transfer computing data through a low-speed PCI-E bus, and the number of work-items started is not enough to hide the time overhead of data transfer and kernel function startup, that is, the performance improvement brought by many-core computing cannot offset the additional communication and function startup time overhead brought by heterogeneous architecture. With the increase of image resolution, the computation shifts from I/O-intensive to computing-intensive. When the image resolution is less than 2048 × 1536, the speedup of the OCL_Canny parallel algorithm increases faster. However, when the image resolution exceeds 2048 × 1536, the slope of the *S*_*OCL*_(NVIDIA) curve gradually smooths and tends to be stable, and the OCL_Canny parallel algorithm achieves a speedup of 20.68 times.

Table 10 shows the acceleration effect of CUDA_Canny and OCL_Canny parallel algorithms and related literature on three groups of images. As can be seen from the table, when dealing with small images, the acceleration effect of the data in Literature [15] is similar to that of the OCL_Canny parallel algorithm. With the expansion of the image frame, the growth rate of *S*_*CUDA*_ and *S*_*OCL*_(NVIDIA) is faster than that of Literature [15], indicating that the OCL_Canny parallel algorithm is more suitable for the fast processing of large images than Literature [15].

**Table 10:** Comparison of acceleration ratio of related literature.

Image resolution (px)	Speedup		
	Literature [15]	*S*_*CUDA*_	*S*_*OCL*_ (NVIDIA)
256×256	2.00	2.90	3.13
512×512	1.86	8.74	9.37
1024×1024	1.81	11.82	14.32

**Fig 13:**
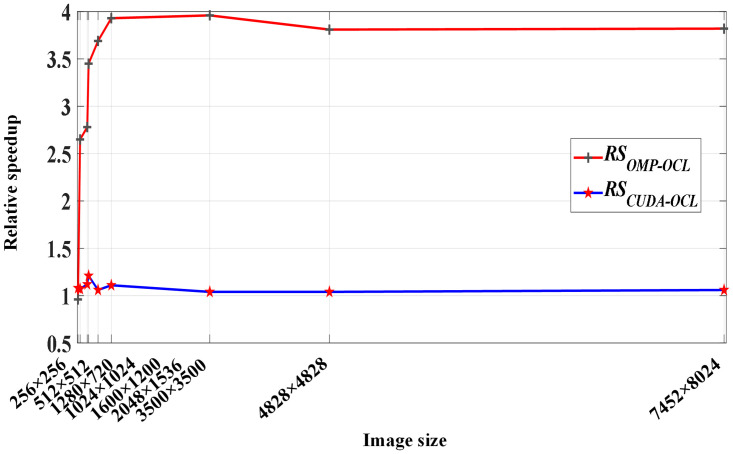
Performance comparison between different parallel Canny algorithms.

Fig 13 visually shows the performance comparison among the three parallel algorithms OMP_Canny, CUDA_Canny, and OCL_Canny. As can be seen from Fig 13, when the image is small, the OCL_Canny parallel algorithm has no obvious performance advantage over the OMP_Canny parallel algorithm. The OCL_Canny parallel algorithm needs data exchange between memory and video memory, which degrades the performance of the OCL_Canny parallel algorithm. However, when the image is larger, the number of work-items started is more, the proportion of kernel function execution time is reduced, and the large value *RS*_*OMP-OCL*_ reflects the strong data processing ability of the GPU. The acceleration ability of the CUDA_Canny and OCL_Canny parallel algorithms is basically the same and *RS*_*CUDA-OCL*_ achieves a maximum acceleration advantage of 1.21 times. This is mainly because the OCL_Canny parallel algorithm adopts the method of offline compiling kernel read and write data files, which reduces the application initialization time compared with the method of online compiling kernel read and write data files by the CUDA_Canny parallel algorithm.

(2) Discussion on portability of the OCL_Canny parallel algorithm

As can be seen from Fig 12, the OCL_Canny parallel algorithm has a good acceleration effect on different GPU platforms. At the same time, the values of *S*_*OCL*_(AMD) and *S*_*OCL*_(NVIDIA) are very similar in nine groups of images with different image sizes. It shows that the OCL_Canny parallel algorithm has good platform scalability and data scalability.

#### 6.3.3. System bottleneck analysis

In the operation and execution of the OCL_Canny algorithm based on GPU acceleration, there are a large number of memory read and write operations in the processing steps of Gaussian filtering, image gradient calculation, image non-maximum value suppression, and edge detection. According to the previous analysis, in the kernel operation of the Gaussian filter, the system needs to read *H*^2^ × *n*^2^ times and write *H*^2^ times to the extended image. In calling the kernel operation to calculate the image gradient, it is necessary to read data *H*^2^ × *n*^2^ times for the extended image and write data 2 × *H*^2^ times for the amplitude and direction of the image gradient. In calling the kernel operation of the non-maximum value suppression of the image, it is necessary to read data 2 × *H*^2^ times for the amplitude and direction of the image gradient and write data *H*^2^ times for the original image. In calling the kernel operation of edge detection, it is necessary to read data *H*^2^ times for the amplitude of the image gradient and write data *H*^2^ times for the original image. Therefore, in the operation and execution of the OCL_Canny algorithm, a total of 2 × *H*^2^ × *n*^2^ + 8 × *H*^2^ memory data are needed to read and write. Suppose, the image resolution is 2048 × 1536, the size of the filter template is 3 × 3, and each pixel takes up 4 B storage space. According to the calculation, the total amount of image data accessed by the OCL_Canny system is about 0.3 GB. The total amount of image data divided by the running time of the kernel 4.81 ms, which shows that the bandwidth of the OCL_Canny system is about 62.37 GB/s. At this point, the actual bandwidth of the system is close to the bandwidth 84 GB/s of GeForce GTX 1050. Therefore, the global memory bandwidth has become the main performance bottleneck of the OCL_Canny system.

## 7. Conclusion

With the rapid development of GPU, GPU is used more and more widely, and the advantage of GPU parallel computing is increasing day by day. At the same time, the requirements for the performance and optimization of parallel computing are getting higher and higher. Through the research on the parallel transplantation and optimization of the Canny edge detection algorithm, this paper puts forward the following three suggestions: (1) For large-scale computing-intensive tasks, the performance of the algorithm can be improved through the parallel computing of the GPU. At the same time, the overall performance can be improved through the cooperation of heterogeneous platforms GPU and CPU. (2) Memory access optimization plays an important role in improving the performance of the overall algorithm. Therefore, the efficiency of memory access can be improved by means of vectorization, data localization, and fine tuning. (3) In order to achieve efficient mapping between threads and the underlying hardware, it is necessary to consider the characteristics of hardware architecture and image processing algorithms, and use several optimization strategies to achieve high-performance algorithms. The experimental results show that the OCL_Canny parallel algorithm achieves a performance speedup of 3.13 times ~ 20.68 times under different image data sizes. It provides a theoretical basis for other image processing algorithms and improves the engineering application value of the image edge detection algorithm.

In the future research, we are going to apply higher performance GPU and CPU to the parallel algorithm of the Canny edge detection. But in fact, when the performance of the GPU has improved significantly, the performance of the CPU should also be improved at the same time. Otherwise, when the performance of the GPU far exceeds that of the CPU, the use of heterogeneous systems will make the operation speed slower than that of GPU systems. This is because high-performance GPU can quickly complete a large number of tasks with high concurrency in a short time, while data transmission tasks can only be performed by CPU serial. When CPU performance is poor, too much time is spent on transferring data and fewer tasks can be assigned to the CPU. Therefore, improving the performance of the CPU will further optimize the data transmission between the host and the device, and make GPU work at full load, which will greatly improve the computing speed of the heterogeneous system.
